# A Metabolomic Approach for Predicting Diurnal Changes in Cortisol

**DOI:** 10.3390/metabo10050194

**Published:** 2020-05-13

**Authors:** Jarrett Eshima, Trenton J. Davis, Heather D. Bean, John Fricks, Barbara S. Smith

**Affiliations:** 1 School of Biological and Health Systems Engineering, Arizona State University, Tempe, AZ 85287, USA; jeshima@asu.edu; 2 School of Life Sciences, Arizona State University, Tempe, AZ 85287, USA; Trenton.J.Davis@asu.edu (T.J.D.); Heather.D.Bean@asu.edu (H.D.B.); 3 Center for Fundamental and Applied Microbiomics, The Biodesign Institute, Arizona State University, Tempe, AZ 85287, USA; 4 School of Mathematical and Statistical Sciences, Arizona State University, Tempe, AZ 85287, USA; jfricks@asu.edu

**Keywords:** volatile metabolomics, cortisol, diurnal, gcxgc-tofms, mental health, predictive modeling, biomarkers, personalized diagnostics

## Abstract

*Introduction:* The dysregulation of cortisol secretion has been associated with a number of mental health and mood disorders. However, diagnostics for mental health and mood disorders are behavioral and lack biological contexts. Objectives: The goal of this work is to identify volatile metabolites capable of predicting changes in total urinary cortisol across the diurnal cycle for long-term stress monitoring in psychological disorders. *Methods:* We applied comprehensive two-dimensional gas chromatography coupled with time-of-flight mass spectrometry to sample the urinary volatile metabolome using an untargeted approach across three time points in a single day for 60 subjects. *Results:* The finalized multiple regression model includes 14 volatile metabolites and 7 interaction terms. A review of the selected metabolites suggests pyrrole, 6-methyl-5-hepten-2-one and 1-iodo-2-methylundecane may originate from endogenous metabolic mechanisms influenced by glucocorticoid signaling mechanisms. *Conclusion:* This analysis demonstrated the feasibility of using specific volatile metabolites for the prediction of secreted cortisol across time.

## 1. Introduction

Cortisol, a glucocorticoid, is a biologically-important steroid hormone with a direct role in the regulation of blood pressure, immune activation, inflammation, energy availability, and metabolism [1]. The synthesis of cortisol, originating in the cortex of the adrenal glands, is a well-understood response to stress and represents the final product of the hypothalamic-pituitary-adrenal (HPA) axis. The predictable daily pattern of cortisol synthesis, known as the diurnal cortisol rhythm, is regulated by a complex network of interacting transcription factors and glucocorticoid-induced signaling cascades (i.e., negative feedback loop) within the HPA axis [2,3]. Studies have long associated the dysregulation of the HPA axis with mental health and mood disorders, with specific emphasis on depression; however, underlying mechanisms still require further elucidation [4,5,6,7]. It has been reported that the hyperactivity of glucocorticoid-receptors (GR) in the hippocampus, common in the dysregulated HPA axis, causes chronic inflammation, reduced GR sensitivity, a lasting decrease in the number of GRs, and cell death [4,8]. Alterations to HPA signaling mechanisms and GR sensitivity over time can impede healthy circadian function, detectable as an increase or decrease in basal cortisol levels, and are frequently reported in studies examining psychiatric disorders [8,9,10].

Common analytical approaches for the clinical quantification of cortisol include immunoassays, gas chromatography mass spectrometry (GC-MS), and liquid chromatography tandem mass spectrometry (LC-MS/MS). Immunoassays remain the method of choice for many clinical laboratories due to throughput-capability, despite limitations in dynamic range and accuracy due to antibody cross-reactions with other endogenous steroids (i.e., prednisolone, corticosterone, and 5-beta-reduced metabolites) [11,12,13]. To overcome limitations in the accuracy, chromatography- and spectrometry-based methods have become increasingly common in clinical laboratories [13]. While published LC-MS/MS methods offer advantages over immunoassay sensitivity, specificity, and cost [14], these approaches often require multi-step sample preparation methods (i.e., liquid extraction or online sample trapping), are slower in sample throughput, and lack standardization across labs [14,15]. Similarly, GC-MS approaches for the direct detection of cortisol are limited by the reduced volatility of steroid hormones and thus require extensive sample pre-processing and derivatization, limiting clinical translation [16]. The application of this volatile metabolomic approach offers advantages over current analytical methods because the identification of metabolites that correlate with cortisol synthesis can be used as putative biomarkers for long-term stress prediction in a point-of-care setting.

Cortisol alone is an insufficient biomarker for the classification of mental health disorders, given that many studies have associated the dysregulation of cortisol with depression, bipolar disorder, schizophrenia, and post-traumatic stress disorder [7,17,18,19]. Despite the recent efforts to better understand the pathophysiology of mental health and mood disorders, a limited number of studies have considered how diurnal changes influence metabolism, and none predict cortisol for applications in long-term, stress-related mental health monitoring [20]. System-level analysis has recently become a promising area of research due to its broader implications for disease presence and progression of highly-phenotypic mental illnesses [21]. Our study aims to elucidate underlying metabolic changes related to stress using untargeted comprehensive two-dimensional gas chromatography coupled with time-of-flight mass spectrometry (GC×GC-TOFMS) sampling of the healthy human urinary volatile metabolome throughout the natural diurnal cortisol cycle. We hypothesize that an underlying relationship between the urine metabolome and diurnal cortisol excretion can be identified by the application of untargeted GC×GC-TOFMS sampling to healthy urine samples, collected across three time points in a single day. The healthy diurnal cortisol rhythm provides an opportunity to measure changes in the urinary metabolome in response to these predictable daily changes for the development of prediction models, with mental health as the motivation for study. This study represents the first attempt, to the best of the authors’ knowledge, at non-invasively predicting changes in cortisol across time using a metabolomic approach for future applications in long-term mental health monitoring and point-of-care diagnostics.

## 2. Results

### 2.1. Multiple Regression and Diagnostics

**Figure 1:**
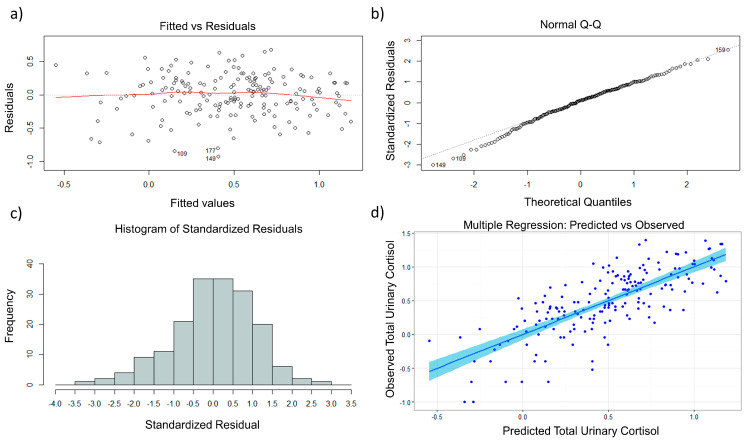
Finalized multiple regression results, using the top 35 variable subset and AICc-guided backward and forward model selection. Both the explanatory and response variables are log_10_ transformed. (**a**) The residual vs. fitted plot supporting the assumption of constant variation in error terms; (**b**) the QQ plot supporting the assumption of error term normality; (**c**) histogram of residuals showing normally distributed error terms; (**d**) predicted vs. observed total free cortisol (in µg) using the multiple regression model with the 95% confidence interval shown in light blue, with values reported as log_10_ transformed.

A volatile metabolomic approach complements clinical translation of mental health diagnostics in a point-of-care setting. HS-SPME-GC×GC-TOFMS analysis allows for the detection of a wide range of volatile organic compounds for use in predicting total free urinary cortisol in urine across the diurnal rhythm. A total of 1551 unique features were initially detected after chromatographic alignment; however, outliers, contaminants, and metabolites that were not present in at least 50% of all samples were removed, resulting in a final dataset containing 512 volatile compounds across 178 healthy urine samples. A bootstrapped elastic-net approach was used to further reduce the number of possible model variables to three smaller datasets containing 25, 35, and 50 VOCs. The reported regression model was identified from the 35-metabolite subset (log-log), utilizing the AICc and BIC metrics, diagnostic plots, adjusted *R*^2^, and the total number of model terms for determination. Table 1 and Table 2 summarize the 14 volatile metabolites and 7 interaction terms (21 total) in the final multiple regression model, respectively. Final model terms were selected using forward and backward stepwise variable selection and minimization of the corrected AIC. Each model term in Table 1 and Table 2 has a reported linear-regression coefficient (*β*_1*,*2*,...,*21_) and 95% confidence interval. Regressor *p*-values were calculated using a t-test, and not all terms were found to be statistically significant; however, manual removal of model terms that were not statistically significant resulted in a decrease in the adjusted *R*^2^ and increase in AICc and BIC metrics, validating the importance of all selected model terms. Regression coefficients for the 14 volatile metabolites indicated that 7 were positively associated and the remaining 7 were negatively associated with measured total urinary cortisol, given that the effects of all other metabolites have been considered. The accompanying multiple regression equation for predicting total urinary cortisol is included, with all metabolite abundances reported as log_10_ transformed and standardized:

Diagnostics [22] were performed to confirm the validity of the model, including: residual vs. fitted plot, QQ plot, residual histogram, residual lag plot, drift plot, variance inflation factor (VIF), added variable plots (Figure S1), and predicted vs. observed plots. The model homoskedasticity can be inferred from the residual vs. fitted plot (Figure 1a). The normality of the standardized residuals can be inferred from the QQ plot (Figure 1b). The normality of the error term distribution can be seen in the residual histogram plot (Figure 1c). Error term independence can be inferred from the plots in Figures S2a and S2b. Overall model performance is displayed as a predicted vs. observed plot, with an adjusted *R*^2^ value equal to 0.521 (Figure 1d). The VIF was calculated for all terms in the multiple regression model to assess the assumption of multicollinearity, and no terms were found to exceed the established cutoff of 5 [22]. In addition, stepwise AICc minimization for the final model was plotted (Figure S2c), but not included in the text.

Figure S3 shows a comparison of model results for the 35 volatile metabolite subset with log_10_ transformation of total urinary cortisol and the 25-metabolite subset without log_10_ transformation of total urinary cortisol. Despite the two models using the same total number of terms (n = 22, including the intercept) and a small difference in adjusted R^2^ values (0.036), the non-linear relationship between explanatory and response variables result in the violation of regression assumptions, underscoring the necessity of the response variable transformation. Additionally, a direct comparison of the six “best” models (top 25, 35, and 50 metabolite sub-sets using raw or log_10_ cortisol values) developed during statistical analysis can be found in Figure S4. It is worth noting that one of the six models (50 metabolite subset with raw values for total free cortisol) was found to have an adjusted *R*^2^ equal to 0.691; however, the model violated assumptions (i.e., linearity, error term normality and heteroskedasticity) and contained 53 total terms, indicating overfitting.

### 2.2. Individual Subject Analysis

**Figure 2:**
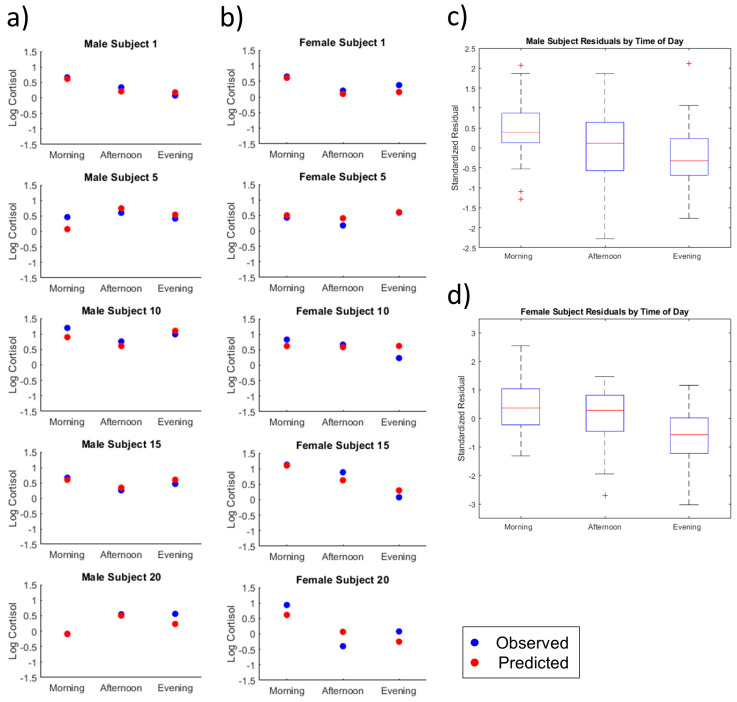
Individual subject analysis by time of day. (**a**) Observed and predicted total free urinary cortisol for five male subjects; (**b**) observed and predicted total free urinary cortisol for five female subjects; (**c**) the boxplot of male subject standardized residuals by time of day; (**d**) the boxplot of female subject standardized residuals by time of day.

Total free urinary cortisol was predicted for each subject using Equation (1). Observed and predicted values were plotted by time of day for all subjects (Figure S5). Representative plots for five male and female subjects were presented in Figure 2a,b, respectively. The authors selected 10 subjects among the 60 participants due to the fact that these plots were indicative of the major predictive trends. It is worth noting that the regression model was able to modestly predict total free cortisol for some of the subjects that did not exhibit typical diurnal shifts (i.e., synthesis of cortisol peaks in the morning followed by a gradual decrease throughout the day), providing additional evidence for the validity of the model, as seen in the plots for male subjects 15 and 20 and female subjects 5 and 20 in Figure 2.

(1) $$\begin{array}{ll} \log10\left(\mathrm{Cortisol}\right)\; & =0.496-0.116\;\times \;1+0.170\;\times \;2+0.035\;\times \;3-0.069\;\times \;4-0.112\;\times \;5+0.207\;\times \;6\\ & -0.040\;\times \;7-0.115\;\times \;8-0.035\;\times \;9+0.004\;\times \;10+0.070\;\times \;11\;-0.063\;\times \;12\\ & +0.070\;\times \;13+0.081\;\times \;14-0.121\;\left(\times 2\times \;\mathrm{Male}\right)-0.100\;(\times 9\times \;\mathrm{Male})\\ & +0.121\;\left(\times 10\times \;\mathrm{Male}\right)+0.210\;\left(\times 3\times \;\mathrm{Morning}\right)-0.295\;(\times 6\times \mathrm{Morning})\\ & -0.075\;\left(\times 14\times \mathrm{Morning}\right)-0.092\;\left(\times 6\times \mathrm{Afternoon}\right) \end{array}$$

#### 2.2.1. Time of Day

Boxplots of standardized residual values by time of day for male and female subjects are presented in Figure 2c–d, respectively. Both male and female subjects displayed similar trends in residuals by time of day, with predicted evening cortisol values being more accurate than morning cortisol values.

For male subjects, morning residual values (total free urinary cortisol) ranged from -0.408 to 0.650 (0.391 µg to 4.47 µg), with an average of 0.140 ± 0.072 (1.38 ± 1.18 µg). Afternoon residual values ranged from −0.803 to 0.557 (0.157 µg to 3.61 µg) with an average of −0.004 ± 0.093 (0.991 ± 1.24 µg). Evening residual values ranged from −0.528 to 0.681 (0.297 µg to 4.80 µg) with an average of −0.063 ± 1.22 µg).

For female subjects, morning residual values ranged from −0.401 to 0.637 (0.397 µg to 4.34 µg) with an average of 0.105 ± 0.086 (1.27 ± 1.22 µg). Afternoon residual values for female subjects ranged from −0.848 to 0.451 (0.142 µg to 2.82 µg) with an average of 0.029 ± 0.096 (1.07 ± 1.25 µg). Evening residual values ranged from −0.929 to 0.330 (0.118 µg to 2.14 µg) with an average of −0.202 ± 0.094 (0.628 ± 1.24 µg).

#### 2.2.2. Male Versus Female

Overall analysis by subject sex revealed the model slightly outperformed for the female subgroup compared to the male subgroup. The average residual value for all female samples was equal to 0.589 µg, whereas the average residual for all male samples was found to be 1.70 µg. In general, the model demonstrates consistent predictive power across sample subgroups (i.e., sex and time of day).

### 2.3. Review of Selected Metabolites

**Figure 3:**
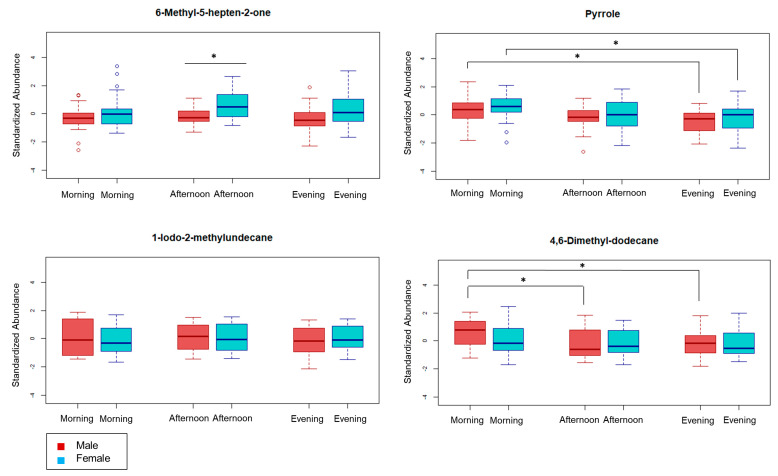
Putatively identified metabolites are presented as boxplots by time of day. Unpaired t-tests were applied to identify statistical significance across all sample subgroups. Statistical significance after an FDR correction is denoted with *.

Volatile metabolites used in the multiple regression model were subject to further statistical analysis using unpaired t-tests. A false discovery rate (FDR) of 0.10 was applied to control for type I errors during multiple hypothesis testing, and five comparisons were found to retain significance. The four metabolites with an ID level ≤ 3 were chosen for representation as boxplots in Figure 3. The abundance of 6-methyl-5-hepten-2-one (sulcatone) was significantly elevated in females for the afternoon subset, suggesting specific volatile metabolites are dependent on sex. Two of the remaining metabolites shown in Figure 3, pyrrole and 4,6-dimethyl-dodecane, demonstrating a time-dependent component to detectable urinary abundance for some metabolites. This relationship suggests that normal, daily biological changes (i.e., diurnal rhythm) influence downstream metabolic pathways, similar to reports from previous studies [23,24,25,26]. Analysis for all 14 volatile metabolites can be found in Figure S6. Additionally, volatile metabolites in the multiple regression model with an ID confidence level ≤ 3 were reviewed for biological context.

## 3. Discussion

Dysregulation of metabolic pathways caused by disease can be detected as changes in metabolite abundance using a variety of analytic techniques including but not limited to LC-MS, GC-MS, and NMR. The ability to identify and quantify metabolites in a biological sample using chromatography and mass spectrometry has generated interest in developing non-invasive and clinically-translatable panels of biomarkers for use in psychiatric disorder phenotyping, early diagnosis, and treatment selection [27,28]. Studies applying volatile metabolomic approaches have demonstrated successes in identifying novel markers of disease including cancer, diabetes, and mental illness [28,29,30].

In this study, urine samples were collected at three time points in a single day for 60 subjects in an effort to probe the underlying temporal relationship between diurnal cortisol levels and excreted metabolites. Using the multiple regression model that we developed, the average female residual value across all samples was found to equal 0.589 µg, while the average residual for all male samples was found to equal 1.70 µg. Through this work, we have shown that the model detailed in Equation (1) can be applied to approximate total free urinary cortisol excretion across time in healthy individuals. The selected multiple regression model includes 14 volatile metabolite terms and 7 interaction terms using factors identified by the two-way ANOVA (i.e., sex and sample collection time). From a biological standpoint, the interaction terms in the model account for variability in metabolite abundance due to sample-specific characteristics (i.e., subject sex and sample collection time). Model diagnostics shown in Figure 2 and Figures S1–S4 provide evidence that underlying regression assumptions of linearity, normality of error terms, multicollinearity, heteroskedasticity, and autocorrelation are satisfied. Diagnostics were included to demonstrate the validity in applying the multiple regression model to approximate total urinary cortisol in the new sample population.

Elevated hydroxyhemopyrrolin-2-one (HPL) in urine has long been associated with psychiatric disorders, first recorded in schizophrenic patients, with similar findings regarding bipolar disorder, depression, hyperactivity disorder, Down Syndrome, and chronic fatigue syndrome [31,32]. Pyrrole (Figure 3), a precursor to HPL, is known to play a role in some biological pathways, including porphyrin synthesis for heme and cytochrome macromolecules. A relevant study by McGinnis et al. suggested an altered heme biosynthesis pathway can produce HPL and other precursor molecules (i.e., pyrrole) after interactions with the gut microbiome [33]. Furthermore, McGinnis et al. found that administration of prednisone, a corticosteroid, caused a statistically significant increase of HPL in urine due to stress-related changes in intestinal permeability, directly linking cortisol signaling mechanisms and metabolite abundance in urine. Interactions with the microbiome may also explain the detection of 6-methyl-5-hepten-2-one (Figure 3), with possible endogenous sources including: (i) the geraniol and nerol degradation pathway for monoterpene metabolism in bacteria [34]; and (ii) oxidative degradation of squalene in bacteria [35,36]. Although 6-methyl-5-hepten-2-one is not a primary metabolite in humans and is used as a synthetic flavoring compound, previous studies have shown that several gut microorganisms, including yeast (Candida parapsilosis, Candida boidinii) and bacteria (Klebsiella oxytoca), utilize the geraniol and nerol degradation pathway as a carbon and energy source [37,38,39]. Also, 1-Iodo-2-methylundecane (Figure 3) has been previously reported as a putative estrogen-dependent chemo-signal in mice, with evidence presented for potential interactions with GR signaling pathways [40,41]. While limited information is present regarding the synthesis of 4,6-dimethyl-dodecane, one study examining volatile metabolites in breath samples from head and neck cancer patients identified 4,6-dimethyl-dodecane (Figure 3) in >80% of healthy subjects (n = 15), supporting its use as a model regressor for applications in new healthy cohorts [29].

Dallmann et al. previously demonstrated that the natural circadian rhythm has a direct effect on multiple human metabolic pathways, shown to be independent of sleep and diet [23]. These results provided the foundation for our model development to predict cortisol levels across time using volatile urinary metabolites for future applications in long-term mental health monitoring. Of the four volatiles with an ID level less than or equal to 3, pyrrole and 6-methyl-5-hepten-2-one were concluded to have possible origins in secondary metabolic pathways and interactions with common gut microbes [33,37]. Results from Vanuystel et al. support the hypothesized interaction between urinary metabolite abundance, gastrointestinal permeability, and time-varying, psychologically-induced stress (i.e., synthesis of CRH and cortisol) [42]. While evidence for the endogenous origin of these compounds is presented, a limited number of studies have explored biosynthesis mechanisms for volatile metabolites [43,44], lowering overall confidence in selected pathways.

The human urinary metabolome has been previously characterized in many different studies, yet few have considered the significant effects of temporal changes [23,24,26,45]. While Dallmann et al. and Ang et al. have previously studied the salivary and plasma metabolome across the circadian cycle, their analysis stops short of utilizing their circadian-dependent metabolites to predict biological changes across time. Our development of a multiple regression model to predict cortisol, and implicitly stress, improves upon the interpretation of diurnal cortisol influences on metabolism for wider applications in psychiatric diagnostics and long-term monitoring. In addition, our application of GC×GC-TOFMS instrumentation offers improvements in the overall resolution and identification capabilities as compared to one-dimensional instrumentations [46], complementing an untargeted approach for volatile urinary metabolomic analysis. Orthogonal separation using non-polar and mid-polar columns helps to reduce co-elution of similar compounds resulting in a greater number of detected and quantified metabolites. Future work will apply the multiple regression model for use in mental health diagnostics; however, targeted analysis using a larger number of subjects must be performed to further validate compound identities, prediction capability, and the accuracy of the model.

## 4. Materials and Methods

### 4.1. Subject Recruitment

The volatile analysis performed in this study involved the collection of three different urine samples from 60 healthy male and female subjects (30 male and 30 female), according to the approved IRB. Subjects were recruited at random from Arizona State University, Tempe campus. A screening survey was implemented to assess physical and mental health prior to recruitment. Subjects were asked to self-report on their average and current stress levels, using a 0-10-point scale, with current stress results shown in Table 3. A qualitative stress cutoff > 6 was set to exclude individuals that recently experienced heightened levels of stress in an effort to target the healthy diurnal cortisol rhythm. A score of 6 was selected based on patient comments accompanying their self-reported score. Scores of ≥ 7 were typically accompanied by comments indicating the participants were experiencing stressful events not typically encountered with high frequency, such as multiple exams, difficult classes, and job interviews. To avoid potential outlier cortisol observations, scores ≥ 7 were excluded. Interested participants that had indicated they were currently taking medication were excluded from the study. Women taking birth control were accepted into the study and comprised 13 of the 30 healthy female subjects. Study subjects spanned 18 to 54 years old, averaging 22 years old, with no specific age range established as a study criterion.This study was approved by the Institutional Review Board (IRB) at Arizona State University (IRB 00006010). Written, informed consent was obtained from all individual participants included in the study.

### 4.2. Sample Collection

Subjects accepted into the study provided unique samples in the morning, afternoon, and evening, at similarly-spaced intervals across a single day (5–7 h between collection periods depending on lunch timing; 12 h separated morning and evening samples). Subjects were asked to withhold from food and liquid consumption 2 h and 20 min prior to sample collection, respectively. The food consumption restriction was established based on a foundational study analyzing gastric emptying-time using ^51^Cr release [47]. Results from this study indicated that a time period of 90 minutes was sufficient for gastric emptying in healthy patients. Subjects were given 90 minutes post-waking to provided their first sample, a restriction implemented to target morning first pass urine. Afternoon samples were collected directly before lunch. Evening samples were collected 2 h before dinner (with at least 5 h since the prior sample collection) or 2 h after dinner. Prior to all sample collection, subjects were screened using a self-reported survey to ensure adherence to study criteria (i.e., food, drink, and medication). Collection containers were gently agitated for 10 s, then four 10 mL samples were aliquoted into cryovials and stored at −80 °C until testing. Volatile analysis was completed within three months of the first sample collection date. Urine samples were sent for LC-MS/MS analysis at a commercial lab to quantify total free urinary cortisol (LabXpress, Phoenix AZ), with all values reported in micrograms (µg).

### 4.3. Volatile Extraction (HS-SPME)

Cryovials containing 10 mL of subject urine were thawed at room temperature for 1 h Vials were then inverted five times to promote a homogeneous mixture. Five mL of the urine sample was aliquoted into a 10 mL heat-treated glass vial (Sigma-Aldrich, St. Louis, MO, USA). Screw-tight PTFE/silicone septa (Sigma-Aldrich) were used to seal the sample and generate a sampling headspace.

Volatiles were extracted from the headspace of biological samples using solid-phase microextraction (HS-SPME; 1 cm, divinylbenzene/carboxen/polydimethylsiloxane, 50/30 µm; Sigma-Aldrich). This SPME fiber coating biases towards low molecular weight volatiles; however, the fiber has been previously identified as the most effective coating to maximize urinary volatile headspace extraction [48]. Headspace equilibrium was promoted by agitating the samples at 250 rpm for 5 min at 50 °C prior to inserting the SPME fiber. The literature suggests an agitation time of 4–5 min is sufficient to reach equilibrium for volatile metabolites [49]. Sampling was performed by exposing the SPME fiber to the urine headspace for 45 min during agitation at 250 rpm and heating at 50 °C. SPME fibers were heated for 5 min at 270 °C between samples to minimize analyte carryover. SPME fibers were replaced every 60 injections to improve sample reproducibility.

### 4.4. GC×GC-TOFMS Analysis

Volatile analysis was performed using GC×GC-TOFMS (Pegasus 4D, Leco Corp., St. Joseph, MI, USA). The first-dimension column consisted of a Rxi-624Sil MS (60 m × 25 µm × 1.4 µm (length × internal diameter × film- thickness); Restek, Bellefonte, PA) and the second-dimension column consisted of Stabilwax (100% polyethylene glycol; 1 m × 25 µm × 0.5 µm); Restek). Detection and data acquisition parameters were controlled using ChromaTOF^®^ software, Version 4.60.8.0 (Leco Corp., St. Joseph, MI, USA). Columns were heated independently using two separate ovens. The primary oven was initiated at 50 °C and held for 2 min, then increased at 5 °C/min until reaching 230 °C and held for 10 min. The secondary oven was maintained at 5 °C above the primary oven. A quad jet modulator was used with a 2 second modulation period. Each modulation period consisted of an alternating 0.5 s hot pulse and 0.5 s cold pulse. The modulator was maintained at a temperature offset of 15 °C relative to the secondary oven. UHP Helium (99.999%) was used as the carrier gas to maintain a flow rate of 1 mL/min. A split injection ratio of 50:1 was used. The inlet and transfer line temperatures were held at 250 °C.

Mass spectra were collected at a rate of 100 Hz over a mass range of 40–500 with an ionization energy of −70 eV. Samples were randomized and urinary volatile metabolite data were collected over three continuous weeks of GC×GC-TOFMS analysis. A perfluorotributylamine (PFTBA) standard was run at the start of each day to tune the MS. Blanks (empty vials) were run daily prior to clinical samples to monitor for system contamination. Alkane standard mix (C_8_-C_20_; Sigma-Aldrich) was sampled at the beginning, middle, and end of the sample set for calculating retention indices.

Instrument methodology was optimized (volume and degradation) for volatile analysis using our previously established approach [50]. In brief, SPME extraction was tested in triplicate for urine volumes of 0.5 mL, 1.0 mL, 2.5 mL, and 5.0 mL, and a maximum number of features identified at 5.0 mL sample volume with statistical significance (*α* = 0.01). Sample degradation was analyzed using a 4 °C temperature-controlled sampling tray. Thirty aliquots (5 mL) of the same urine sample were analyzed by GC×GC-TOFMS, resulting in less than 5% relative standard deviation in the number of detected VOCs. From this preliminary analysis, it was determined that urine samples remained viable for up to 39 hours in the temperature-controlled sampling tray, prior to testing.

### 4.5. Data Processing

Volatile profiles were processed and aligned using the Statistical Compare package within the ChromaTOF software. The baseline signal was drawn through the middle of the noise. S/N used in peak selection was set at 50:1 for a minimum of two apexing masses. Subpeaks in the second dimension were required to meet a S/N ≥ 6, mass spectral match ≥ 600, and a retention time shift ≤ to 0.2 s to be combined within an individual chromatogram. For chromatographic alignment, peaks had to have ≤ 2 second retention time shift in the first dimension and ≤ 0.2 second retention time shift in the second dimension. Additionally, a minimum spectral similarity score of 600 (60%) was required prior to alignment. A secondary round of peak picking was performed during alignment using a S/N threshold of 5. Peaks eluting prior to 358 s, as well as peaks identified in blank samples, were excluded from statistical analysis. Compound abundance was obtained by integrating the areas of aligned peaks using the unique ion mass. The resulting aligned peaks were then compared to the National Institute of Standards and Technology (NIST) 2011 Mass Spectral Library. Tentative peak names were assigned to mass spectra with similarity scores ≥ 600. Analytes with a spectral similarity score <600 were labeled “unknown”. However, tentative names from the instrument library search provide a foundation for targeted method development.

Putative metabolic biomarkers identified by the statistical analysis were assigned ID confidence levels, ranging from 1–4 (1 being most confident), using previously published guidelines established by the Metabolomics Standards Initiative [51]. Compounds that were verified with an ≥80% mass spectral match on a forward search in the NIST 2011 library and with linear retention index (RI) data consistent with the mid-polar Rxi-624Sil stationary phase received a confidence level of 2. Volatile metabolites included in the predictive model with an ID level of 2 were verified by analytical grade standards ≥98% pure (pyrrole, 6-methyl-5-hepten-2-one; Sigma Aldrich) to reduce the confidence level to 1. RIs were calculated using the following equation, with a 5–35% cutoff used for level 2 classification [52]:

Mean RIs for non-polar and polar columns were obtained from the NIST 2011 RI database and published studies [53,54]. Peaks with a spectral match ≥80% but without confirmatory RI (Equation (2)) or homologous series information were assigned an ID confidence level of 3, using NIST non-polar RIs as supporting evidence for naming. Compounds that could not be validated against RI or homologous series data with a spectral match less than or equal to 80% received an ID confidence level of 4. Tentative names assigned to level 4 compounds by the software were replaced with functional group identifiers if second-dimension elution time provided sufficient evidence [55]. Otherwise, level 4 volatile metabolites in Table 1 and Table 2 were reported as “unknown”.

(2) $$\frac{RI_{experimental}-RI_{non-polar}}{RI_{polar}-RI_{non-polar}}*100=5-35\%$$

### 4.6. Statisical Analysis

#### 4.6.1. Post-Processing

Raw metabolite abundances were normalized using Probabilistic Quotient Normalization (PQN) [56]. Abundance values were then log_10_ transformed, centered, and scaled using R, Version 3.3.2 (The R Foundation for Statistical Computing, Vienna, Austria). Compounds that were not present in at least half of all samples were removed (MATLAB 2018b; Mathworks Inc., Natick, MA, USA) in an effort to improve the translatability of the results. Two outliers (male subject 6 morning, 44.3 µg; male subject 29 afternoon, 97 µg) were removed from all post-processing and subsequent statistical analysis, given that the adult reference range for total free urinary cortisol in 24 h is roughly 3.5–45 µg [57]. A two-way ANOVA with replication was performed to determine if sex (*p* = 0.013) and time of day (*p* = 0.034) were statistically significant sources of variation for use as interaction term factors in the multiple regression model. Given that our dataset contains far more variables than observations, the two-way ANOVA and elastic-net were used in an exploratory manner to reduce the total number of variables used in the regression.

#### 4.6.2. Variable Selection

Model variables were selected by applying elastic-net using R package glmnet, Version 2.0-5 [58,59] and bootstrapping to the sample set using 80% of subjects (n = 48) as training and the remaining 20% as the test. An alpha = 0.5 was selected as the weight of the lasso (L1) vs. ridge (L2) optimization. Three subsets of volatile metabolites, containing the top 25, 35, and 50 compounds, were generated using a frequency of selection by elastic-net over 250 iterations using minimization of the mean squared error to guide variable selection. Each subset of metabolites was used to develop a multiple regression model with sex and time of day included as interaction terms. Sex was defined as a binary dummy variable where all male subjects received a 1 and female subjects received a 0, for all time points. The time of day interaction term was split into two dummy variables, in which:

(i) morning samples received a 1 and all other samples received a 0
(ii) afternoon samples received a 1 and all other samples received a 0.

#### 4.6.3. Multiple Regression

**Figure 4:**
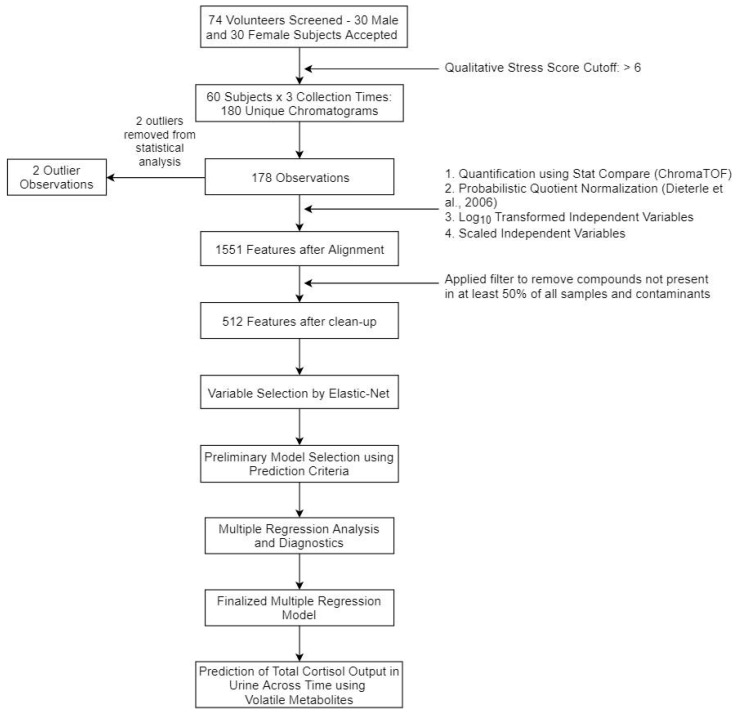
Flowchart showing the process for urinary metabolome identification and statistical analyses.

Initial models for each subset of volatile metabolites (top 25, 35, and 50) contained all possible variables and interaction terms. Model selection was performed using forward and/or reverse stepwise minimization of Akaike and Bayesian Information Criterion (AIC and BIC, respectively) for each of the three metabolite subsets using the MASS R package, Version 7.3-45 [22,60]. A model was selected for each subset using the smallest corrected AIC (AICc) as an initial metric, and the smallest BIC to select between multiple models with similar AICc values, if necessary. Regression diagnostics were then performed to check for model adherence to underlying assumptions. After reviewing the selected models, it was determined that a log_10_ transformation of the response variable (total free cortisol) was necessary. A second round of model development was performed for the top 25, 35, and 50 metabolite subsets yielding the final regression model reported in Equation (2) *p-*values, for model terms were calculated using a t-test and subsequently corrected using the Benjamini-Hochberg adjustment procedure [61]. The applied workflow has been summarized in Figure 4.

## 5. Conclusions

Diagnostics for psychiatric disorders remain qualitative and lack biomarkers due to complex, multi-level system interactions and widely varying phenotypes. This study aims to improve long-term mental health monitoring through the ability to track and predict changes in stress across time. Multiple regression modeling was performed on 60 healthy subjects, resulting in the development of a linear model, with 21 total terms, to predict total excreted cortisol in urine. Through our review of selected metabolites, biosynthesis pathways were identified for compounds with confirmatory chromatographic and mass spectral information. Furthermore, related studies have reported a correlation between pyrrole and diurnal cortisol levels, thus supporting our identification of pyrrole as a potential stress marker in future targeted studies. Results from this study support the potential for developing non-invasive methods to monitor stress across time, using downstream metabolites as predictors.

## Figures and Tables

**Table 1:** Results for individual terms in the multiple regression model. Metabolite information includes: compound name, Human Metabolome Database (HMDB) ID, chemical classification, regression coefficient, confidence interval, t-test corrected *p*-value, 1*^st^* dimension retention time, 2*^nd^* dimension retention time, retention index, and ID confidence level using the guidelines set by the Metabolomics Standards Initiative. Retention indices denoted with * have been extrapolated. Compounds denoted with ** have assigned names other than the first spectral hit, using NIST non-polar RI database for confirmation. Compounds denoted with *** fell within 6% of NIST reported non-polar RI.

Multiple Regression Model–Metabolite Term Breakdown										
Model Variable	Compound Name	HMDB ID	Chemical Classification	Regression Coefficient	95% Confidence Interval	Benjamini-Hochberg Adjusted *p*-Value	^1^t_R_(s)	^2^t_R_(s)	RI	ID
β_0_	Intercept	NA	NA	0.496	(0.446, 0.548)	< 2 × 10^−16^	NA	NA	NA	NA
x_1_	6-Methyl-5-hepten-2-one	HMDB0035915	Ketone	−0.116	(−0.174, −0.058)	2.93 × 10^−6^	1364	0.76	1031	1
x_2_	Ketone 1	‒	Ketone	0.170	(0.093, 0.247)	4.18 × 10^−8^	2056	0.75	1403	4
x_3_	Unknown 1	‒	‒	0.035	(−0.048, 0.117)	2.16 × 10^−8^	1102	0.94	911	4
x_4_	Hydrocarbon 1	‒	Hydrocarbon	−0.069	(−0.127, 0.012)	2.70 × 10^−3^	1926	0.57	1326	4
x_5_	Unknown 2	‒	‒	−0.112	(−0.169, −0.055)	4.57 × 10^−3^	1510	1.03	1102	4
x_6_	Unknown 3	‒	‒	0.207	(0.105, 0.308)	4.79 × 10^−3^	2270	1.00	1538	4
x_7_	Unknown 4	‒	‒	−0.040	(−0.095, 0.014)	4.08 × 10^−2^	512	1.47	644 *	4
x_8_	Unknown 5	‒	‒	−0.115	(−0.173, −0.057)	6.37 × 10^−2^	918	0.64	830	4
x_9_	Unknown 6	‒	‒	−0.035	(−0.112, 0.042)	6.80 × 10^−2^	2038	0.52	1392	4
x_10_	Unknown 7	‒	‒	0.004	(−0.095, 0.104)	1.57 × 10^−2^	2156	0.54	1456	4
x_11_	Pyrrole	HMDB0035924	Heteroaromatic	0.070	(0.001, 0.139)	1.39 × 10^−1^	926	1.87	833	1
x_12_	1-Iodo-2-methylundecane **	HMDB0062727	Halogenated Hydrocarbon	−0.063	(−0.124, −0.003)	5.47 × 10^−2^	2322	0.59	1571	3
x_13_	Unknown 8	‒	‒	0.070	(0.003, 0.137)	8.97 × 10^−2^	2304	1.07	1560	4
x_14_	4,6-Dimethyl-dodecane ***	HMDB0062598	Hydrocarbon	0.081	(0.001, 0.161)	8.97 × 10^−2^	1784	0.56	1246	3

**Table 2:** Interaction terms used in the model have the following information provided: compound name, dummy variable, regression coefficient, confidence interval, and F-statistic corrected *p*-value. Compounds denoted with *** fell within 6% of NIST reported non-polar RI.

Multiple Regression Model–Interaction Term Breakdown					
Interaction Terms	Compound Name	Dummy Term	Regression Coefficient	95% Confidence Interval	Benjamini-Hochberg Adjusted *p-*Value
x_2_ × Male	Ketone 1	Male	−0.121	(−0.228, −0.013)	1.81 × 10^−2^
x_9_ × Male	Unknown 6	Male	−0.100	(−0.203, 0.005)	3.18 × 10^−2^
x_10_ × Male	Unknown 7	Male	0.121	(0.004, 0.237)	7.41 × 10^−3^
x_3_ × Morning	Unknown 1	Morning	0.210	(0.076, 0.345)	6.82 × 10^−2^
x_6_ × Morning	Unknown 3	Morning	−0.295	(−0.434, −0.155)	1.25 × 10^−1^
x_14_ × Morning	4,6-Dimethyl-dodecane ***	Morning	−0.075	(−0.182, 0.031)	1.91 × 10^−1^
x_6_ × Afternoon	Unknown 3	Afternoon	−0.092	(−0.218, 0.035)	1.80 × 10^−1^

**Table 3:** Demographics of healthy subjects. Included are subject age, ethnicity, height, weight, and qualitative stress score (at the time of subject consent). Women taking birth control are denoted with *.

Male Subject	Age (years)	Ethnicity	Height (cm)	Weight (kg)	Stress Score (0–10)	Female Subject	Age (years)	Ethnicity	Height (cm)	Weight (kg)	Stress Score (0–10)
1	18	Caucasian	180	64	3	1	26	Latino	163	56	0
2	18	Caucasian	168	59	3	2	18	Asian	165	51	4
3	28	Asian	178	62	5	3 *	20	Latino	157	43	4
4	19	African American	185	95	3	4	23	Caucasian	163	59	2
5	26	Asian	178	50	4	5	18	Asian	163	45	6
6	18	Caucasian	183	79	0	6	19	Caucasian	178	64	5
7	21	Asian	180	75	0	7 *	21	Caucasian	163	54	4
8	22	Caucasian	179	74	0	8 *	32	African American	160	75	4
9	20	Caucasian	170	70	2	9	18	Latino	165	54	3
10	20	Asian	152	50	4	10*	25	Latino	170	63	3
11	20	Asian	173	52	1	11	28	Caucasian	170	67	3
12	19	Latino	170	82	0	12	44	Asian	165	58	2
13	20	Latino	183	73	6	13	30	Latino	163	51	2
14	21	Caucasian	180	84	2	14	18	Caucasian/ Latino	170	82	3
15	18	Latino	160	61	2	15 *	20	Caucasian	170	66	5
16	22	Asian	179	70	0	16 *	19	Latino	173	75	6
17	18	Caucasian	180	68	3	17 *	25	African American	168	70	4
18	20	Caucasian	183	86	2	18	23	African American	165	53	2
19	18	Caucasian	183	68	6	19	22	Caucasian/ Asian	157	61	6
20	21	Caucasian	178	68	4	20	54	Caucasian	157	51	0
21	23	Caucasian	193	77	4	21	20	African American	165	62	0
22	18	Asian	173	73	4	22	21	Caucasian	170	73	6
23	26	Indian	170	70	6	23	18	Latino	157	48	4
24	20	Asian	178	77	6	24 *	18	Caucasian/ Asian	165	59	6
25	19	Latino	178	77	4	25 *	21	Caucasian	168	64	6
26	22	Asian	183	84	3	26 *	19	Caucasian	157	68	3
27	22	Caucasian	183	77	0	27 *	23	African American	157	52	2
28	20	Asian	175	79	0	28	23	African	158	55	5
29	18	Asian	170	61	4	29 *	18	Caucasian	160	64	5
30	18	Asian	185	77	5	30 *	21	Latino	160	52	4

## Data Availability

The datasets generated and analyzed in this study are available in the MetaboLights repository, www.ebi.ac.uk/metabolights/MTBLS1206 [62].

## Supplementary Materials

The following are available online at https://www.mdpi.com/2218-1989/10/5/194/s1, Figure S1: Add variable plots for terms in the finalized multiple regression model. Figure S2: Additional diagnostics plots including the residual lag plot, drift plot, and a plot of stepwise AIC-minimization. Figure S3: Secondary regression analysis to verify the need for response variable transformation. Figure S4: Top 6 regression models constructed during statistical analysis, used to justify final model selection. Figure S5: Individual subject predictions using the predictive regression model. Figure S6: Boxplots of metabolite abundance values used in the model, including pairwise t-testing for significance by sex and time of day.
