# Supplementary material for: A Metabolomic Approach for Predicting Diurnal Changes in Cortisol
Source: Metabolites. 2020 May 13;10(5):194. doi: 10.3390/metabo10050194 (PMC7281277; doi:10.3390/metabo10050194)

**Supplemental Information**

**A Metabolomic Approach for Predicting Diurnal Changes in Cortisol**

**Jarrett Eshima ^1^, Trenton J. Davis ^2,3^, Heather D. Bean ^2,3^, John Fricks ^4^ and Barbara S. Smith ^1,^***

^1^ School of Biological and Health Systems Engineering, Arizona State University, Tempe, AZ, 85287, USA, jeshima@asu.edu

^2^ School of Life Sciences, Arizona State University, Tempe, AZ, 85287, USA, tjdavi18@asu.edu (T.J.D.); Heather.D.Bean@asu.edu (H.D.B.)

^3^ Center for Fundamental and Applied Microbiomics, The Biodesign Institute, Arizona State University, Tempe, AZ, 85287, USA

^4^ School of Mathematical and Statistical Sciences, Arizona State University, Tempe, AZ, 85287, USA, jfricks@asu.edu

***** Correspondence: BarbaraSmith@asu.edu; Tel.: +1-(480)-727-8988


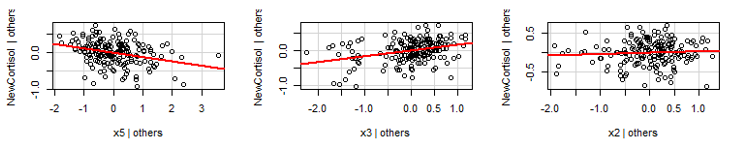

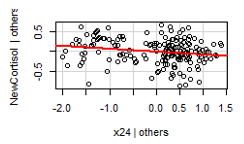

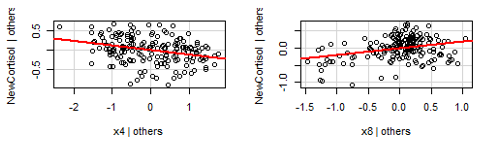

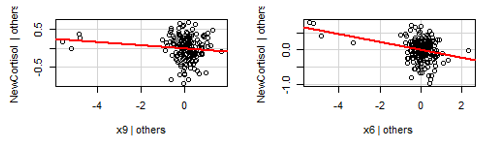

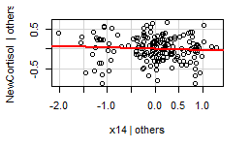

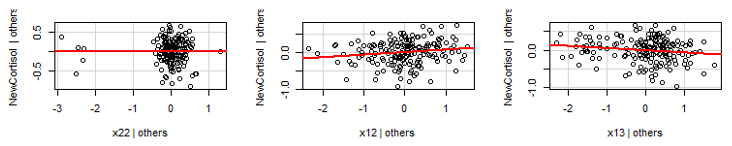

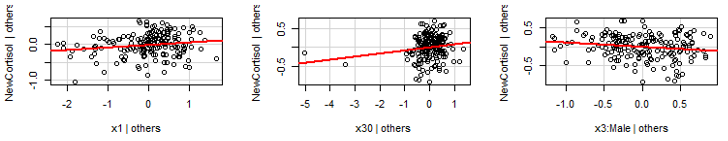

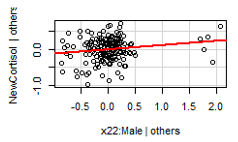

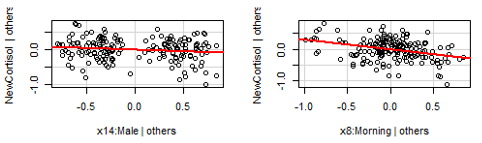

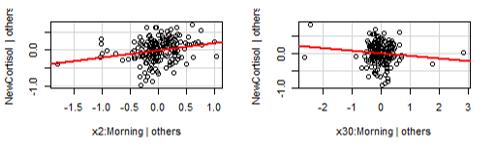

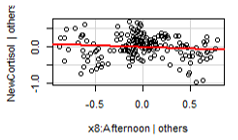


**Figure 1.** Added variable plots showing residual cortisol values for the range of observed abundances in each model term, where all other terms are held constant. These plots show the association (positive, negative, or none) between a single model regressor and the response variable. Plots are presented in the same order as the terms listed in Table 2. Values are reported as log_10_.


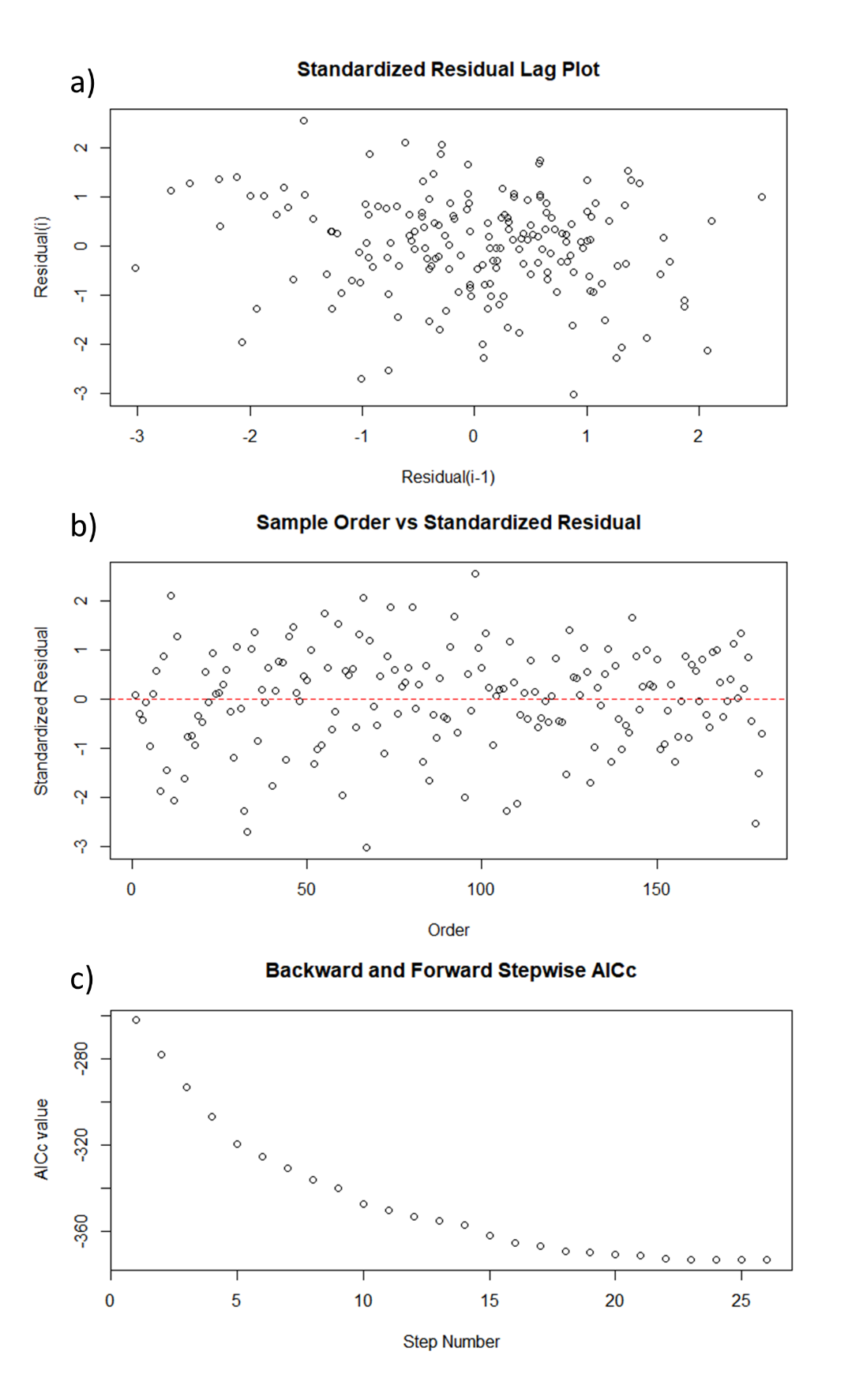


**Figure 2.** Additional diagnostics performed for the multiple regression analysis: a) the residual lag plot validating the assumption of error term independence; b) the drift plot displaying the standardized residual values across the sample run order providing additional support for the assumption of error term independence; and c) the AIC-minimization plot from the stepwise model selection.


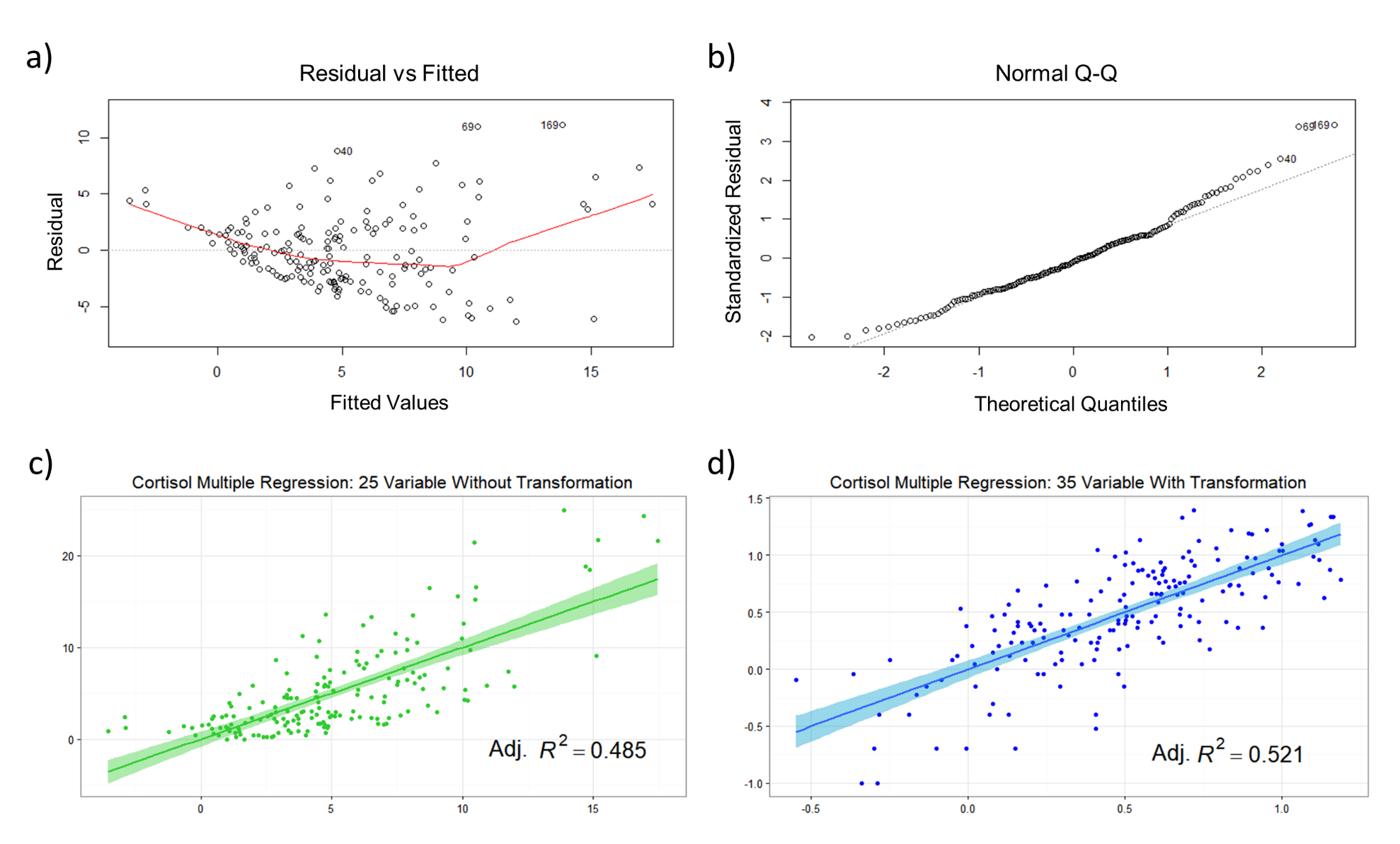


**Figure 3.** Secondary regression analysis to validate the need for the response variable transformation, a) residual versus fitted plot for the model using raw cortisol (reported in µg) suggests non-linearity and heteroskedasticity, b) QQ plot for the model using total urinary cortisol indicating the standardized residuals are not normally distributed, c) predicted versus observed plot for the model using total raw urinary cortisol (reported in µg), and d) final model selected after information metrics and diagnostics using log_10_ transformed total urinary cortisol.


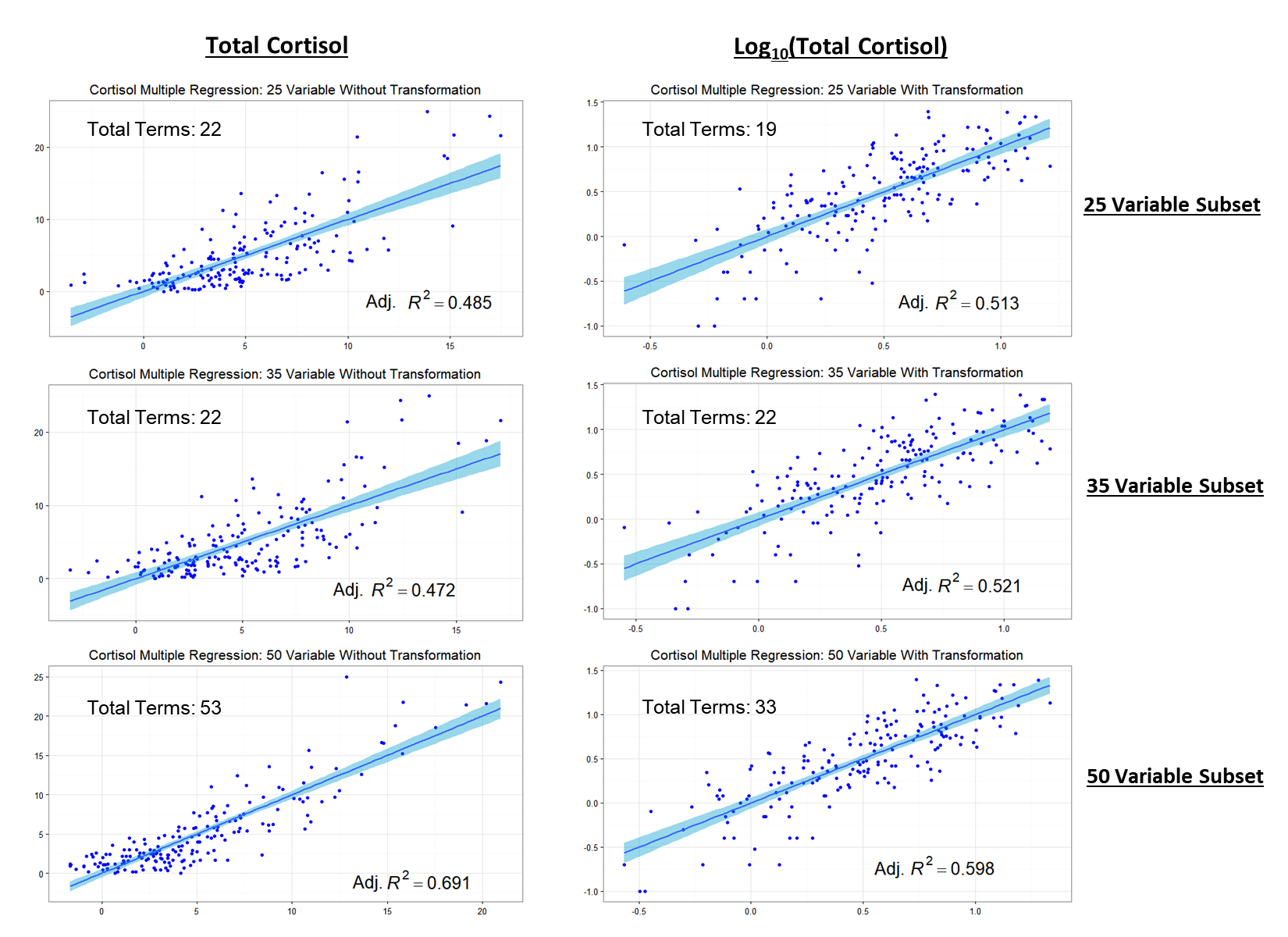


**Figure 4.** The top 6 multiple regression models with predicted urinary cortisol values plotted on the x-axis and observed urinary cortisol values plotted on the y-axis comparing (i) the number of starting variables used in model development, and (ii) the transformation of the response variable (i.e. total urinary cortisol). All metabolite values have been log_10_ transformed in the above models. Included on each observed versus predicted plot is the total number of model terms, identified by summing the number of individual covariates and interaction terms.


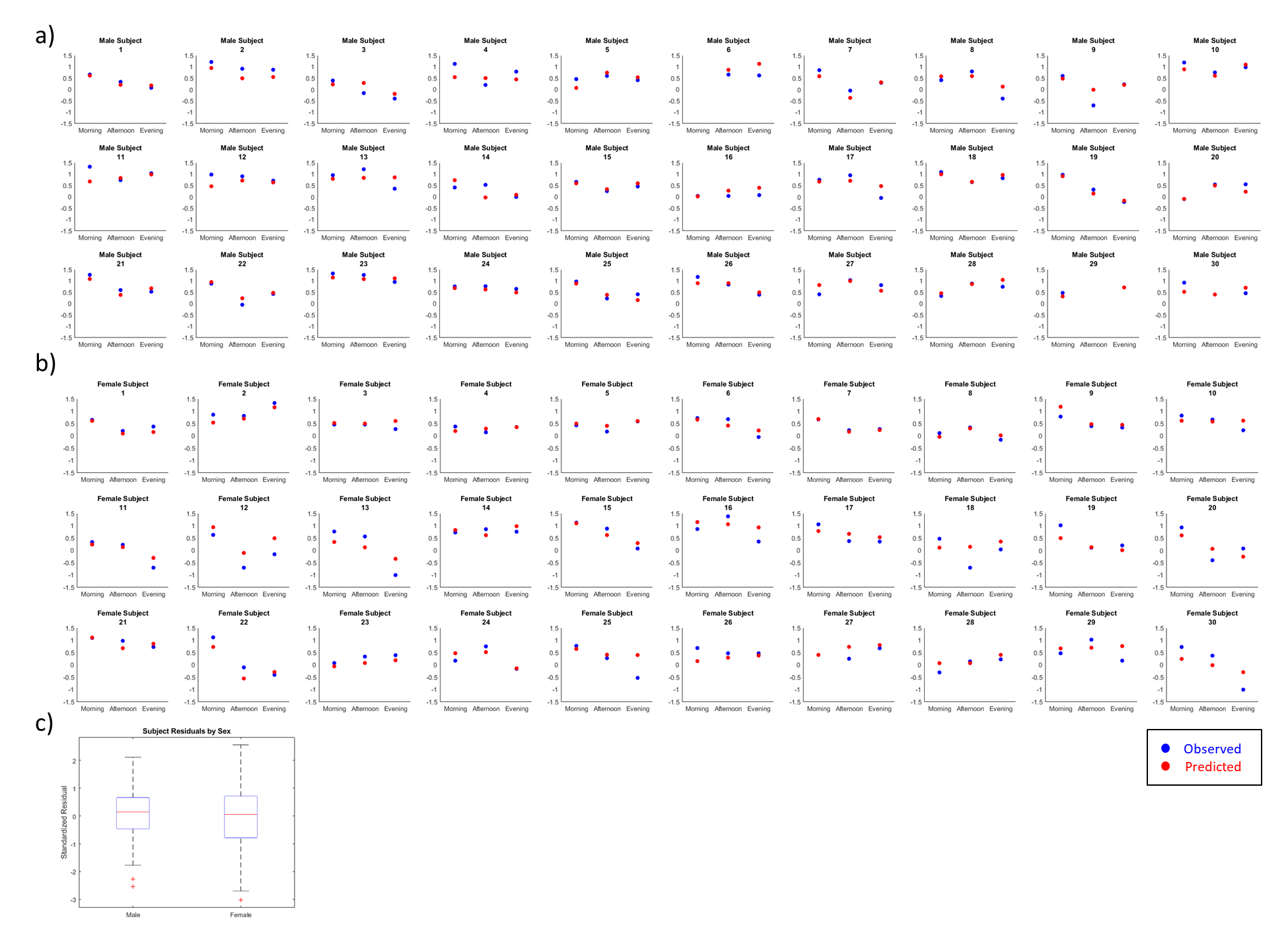


**Fig S5.** Individual subject analysis by time of day (x-axis) with respect to log_10_ cortisol values (y-axis), a) observed and predicted total urinary cortisol for all 30 male subjects, b) observed and predicted total urinary cortisol for all 30 female subjects, and c) boxplot of standardized residuals by sex. Residual values are roughly centered around 0.

**Figure 6.** Metabolite boxplots by time of day and sex. Standardized abundance is plotted on the y-axis. Unpaired t-tests were applied to identify statistical significance across sample subgroups. Statistical significance is denoted with (*) after a False Discovery Rate correction using 0.1 as the proportion of false discovery.


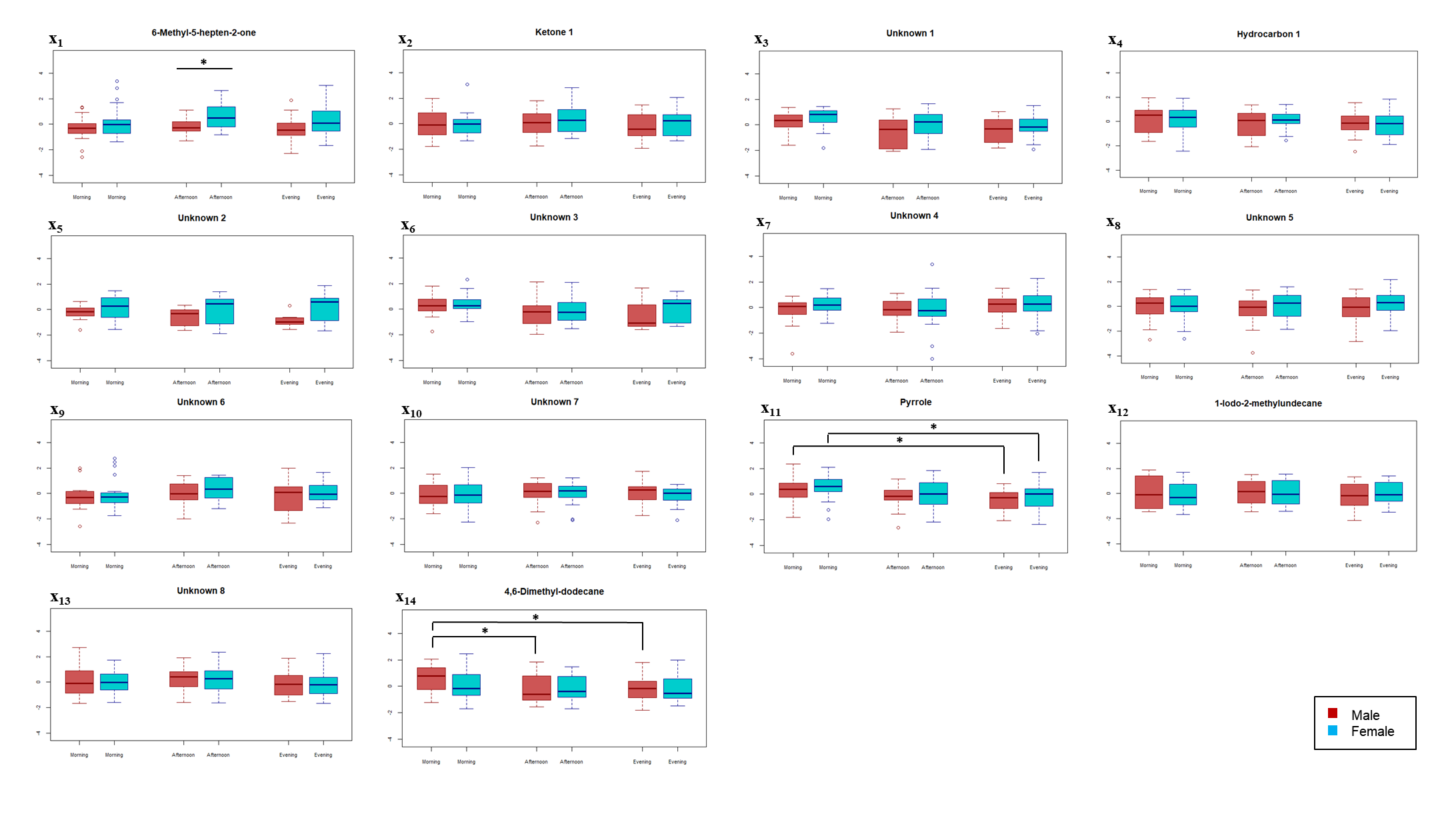

Supplement: Supplementary file 1 [file metabolites-10-00194-s001.zip › metabolites-755031-supplementary.docx]
